# Evaluation and comparison of in vitro antioxidant activities of unsaponifiable fraction of 11 kinds of edible vegetable oils

**DOI:** 10.1002/fsn3.823

**Published:** 2018-10-11

**Authors:** Sujun Wang, Ruinan Yang, Hui Li, Jun Jiang, Liangxiao Zhang, Qi Zhang, Peiwu Li

**Affiliations:** ^1^ Oil Crops Research Institute Chinese Academy of Agricultural Sciences Wuhan China; ^2^ Key Laboratory of Biology and Genetic Improvement of Oil Crops Ministry of Agriculture and Rural Affairs Wuhan China; ^3^ Quality Inspection and Test Center for Oilseed Products Ministry of Agriculture and Rural Affairs Wuhan China; ^4^ Key Laboratory of Detection for Mycotoxins Ministry of Agriculture and Rural Affairs Wuhan China; ^5^ Hubei Collaborative Innovation Center for Green Transformation of Bio‐Resources Wuhan China; ^6^ Laboratory of Risk Assessment for Oilseeds Products (Wuhan) Ministry of Agriculture and Rural Affairs Wuhan China

**Keywords:** comparison, edible vegetable oils, evaluation, extracts, radical scavenging capabilities

## Abstract

The radical scavenging capabilities of the extracts from eleven edible vegetable oils were investigated by using 2,2‐diphenyl‐1‐picrylhydrazyl (DPPH), 2,2′‐azino‐bis‐3‐ ethylbenzothiazoline‐6‐sulfonic acid (ABTS), and ferric reducing ability of plasma (FRAP) assays. The results indicated that rapeseed oil and sesame oil showed higher radical scavenging abilities than other vegetable oils. When the radical scavenging capabilities of the extracts from virgin camellia oils and commercially available refined camellia oils were evaluated by FRAP assay, the results showed that the antioxidant capabilities of the former were higher than the latter. Therefore, it is recommended that moderate refining processes should be taken to minimize the loss of antioxidant components and people consume virgin oils or less processed edible vegetable oils for higher antioxidant activities.

## 1 INTRODUCTION

Highly active free radicals (FRs) are generated from normal and essential metabolic processes or derived from external sources such as air pollutants and industrial chemicals. If excessive FRs exist, oxidant reactions may be induced and cause many diseases, such as cancers, autoimmune disorders and Alzheimer's and Parkinson's diseases (Bagchi et al., 2000; Devasagayam et al., 2004; Lobo, Patil, Phatak, & Chandra, 2010). Antioxidants can effectively scavenge FRs so as to decelerate human aging and prevent diseases. Lipid oxidation may also be induced by FRs, which may lead to bioactive nutrient loss and deteriorate the quality of edible vegetable oils (Bagchi et al., 2000; Castelo‐Branco, Santana, Di‐Sarli, Freitas, & Torres, 2016). Therefore, it is necessary to assess the radical scavenging capabilities of antioxidant active compounds in edible vegetable oils, which are essential in human daily life.

Edible vegetable oils contain a variety of nutritional components, such as phytosterols, tocopherols, and polyphenols (Yang et al., 2018). The radical scavenging capabilities of these bioactive ingredients have become hot research topics in recent years, and studies have showed that these nutritional components could provide antioxidant capabilities (Kris‐Etherton et al., 2002). Gao (2009) investigated the antioxidant effects of different amounts of brassicasterol under high temperature after it was added to sunflower seed oils. It was found that brassicasterol in sunflower seed oil had antioxidant effects and an increased dose produced a more noticeable effect. β‐carotenoid was found to inhibit the oxidation of rapeseed oil and could work with tocopherols to achieve higher antioxidative activities (Xu, Li, & Zhang, 1999). Xu, Hua, and Godber (2001) investigated the antioxidant activities of tocopherols, tocotrienols, and gamma‐oryzanol in a cholesterol oxidation system and found that all these compounds exhibited notable antioxidant activities.

It is difficult for animal models to comprehensively reveal the total antioxidant capability due to their high cost and low usability in antioxidant capability measurements. In vitro antioxidant reactions to evaluate antioxidant activities of food are simple and rapid and therefore widely used. At least two different methods should be used to assess the antioxidant capability of a target since no standard in vitro antioxidant evaluation methods are available. All of the 2,2′‐azino‐bis(3‐ethylbenzothiazoline‐6‐sulfonic acid) (ABTS), 2,2‐diphenyl‐1‐ picrylhydrazyl (DPPH), and ferric reducing ability of plasma (FRAP) assays were used in this work based on the concentrations of trace bioactive materials in edible vegetable oils to study the systematic antioxidant activities of these oils. Recently, the antioxidant capabilities of the edible oils including virgin coconut oil (Ghani et al., 2018), virgin olive oil (Xiang et al., 2017), sesame oil (Saleem, Chetty, & Kavimani, 2013), and sunflower oil (Aladedunye, Przybylski, Niehaus, Bednarz, & Matthäusa, 2014). It seems that edible oils possess more or less antioxidant capabilities. To discover the advantage of edible oils, it is necessary to detect the antioxidant capabilities of edible oils and compare them with each other. The results in this study reflect the nutritional values of 11 edible oils, which would help consumers comprehensively evaluate the quality and nutritional functions of edible oils.

## 2 MATERIALS AND METHODS

### 2.1 Chemicals and reagents

HPLC‐grade methanol and acetonitrile, analytical‐grade acetone, ether, petroleum ether, n‐hexane, and the FRAP kit were supplied by Sinopharm Chemical Reagent Co., Ltd (Shanghai, China). Deionized water (resistivity: 18.2 MΩ cm at 25°C) was obtained from a Milli‐Q water filtration system purchased from Millipore Co., Ltd (Milford, MA, USA). Ethanol was obtained from Xilong Chemical Co., Ltd (Shantou, China). The DPPH and ABTS kits were purchased from Baomanbio (Germany) and Fisher Scientific (USA), respectively. Formic acid (purity ≥95%) was obtained from Sigma (USA).

### 2.2 Apparatus

A refrigerator was bought from Siemens (Germany). Spectra Max M microplate reader was obtained from Molecular Devices in the United States. A constant temperature incubator and Nunc‐Immuno 96‐well plates were supplied by Thermo Fisher (USA). A micropipette and CPA224S electronic analytical balance were purchased from Eppendorf (Germany) and Sartorius (Germany), respectively. Graduated flasks and beakers were from Tianjin Glass Instrument Factory (Tianjin, China). A vortex mixer was obtained from Beijing North TZ‐Biotech Development Co., Ltd. (Beijing, China).

### 2.3 Test sample preparations

Flaxseed oil, olive oil, grape seed oil, corn oil, soybean oil, sunflower seed oil, walnut oil, perilla oil, rapeseed oil, sesame oil, and camellia oil were numbered in the laboratory and stored in the dark. Camellia oils numbered 1–12 were self‐prepared, and No. 13‐39 were purchased from the market.

A total of 0.2 g (accurate to 0.005 g) vegetable oil was weighed and placed into a glass tube with a plug. A volume of 6 ml 2 mol/L KOH ethanol solution was added for dissolution and evenly mixed by a liquid mixer for 1 min. Saponification was performed in an ultrasonic bath with the ultrasonic conditions as follows: temperature: 75°C; time: 40 min; power: 300 W.

After saponification, the sample was cooled at room temperature and then 7 ml distilled water and n‐hexane (1:1, v/v) were added. The unsaponifiable matter was extracted three times with 3.5 ml hexane. The combined hexane fractions were washed 2‐4 times with 10% ethanol/water (v/v) until the washing solution was neutral. The hexane phase was evaporated under a stream of nitrogen, and the residue was dissolved in 1 ml methanol and filtered through a 0.22 μm membrane filter. Subsequently, the obtained liquid was stored at −20°C in a refrigerator for later use. The measurements were performed three times to obtain average values.

### 2.4 Measurements of radical scavenging capabilities

#### 2.4.1 Measurement of radical scavenging capabilities by DPPH assay

The experiment was carried out according to the report of Brand‐Williams (Brand‐Williams, Cuvelier, & Berset, 1995) with slight modification. Before the experiment, the reagents saved in the refrigerator at −20°C were thawed under room temperature. A volume of 5 ml GENMED dye liquor B (Reagent C2) was added to a bottle filled with GENMED dye liquor A (Reagent C1), and then the mixture was vortexed evenly and labeled as GENMED dye working solution. It should be noted that the GENMED dye working solution must be saved in the dark before use.

The standard curve solution was prepared as follows: Five centrifugal tubes (1.5 ml) were labeled 1–5. GENMED buffer (Reagent B) and GENMED standard solution (Reagent D) listed in Table 1 were added into each tube, shaken thoroughly, and then stored in the dark.

**Table 1:** Preparation of standard solutions for measurements of radical scavenging capabilities by DPPH assay

Number	1	2	3	4	5
GENMED buffer (Reagent B, μl)	0.0	20.0	40.0	60.0	100.0
GENMED standard solution (Reagent D, μl)	100.0	60.0	40.0	20.0	0.0
Trolox standard concentration	300.0	240.0	180.0	120.0	0.0

The measurement was performed in the following sequence: (a) Label the blank control, standard and sample wells in a 96‐well plates; (b) Use a micropipet to dispense 205 μl GENMED buffer (Reagent B) into each well; (c) Add 25 μl GENMED dye working solution to each well; (d) Add 20 μl GENMED buffer (Reagent B) into blank control wells; (e) Add 20 μl GENMED standard solution (Reagent D) into wells and add 20 μl sample into sample wells; (f) Shake the 96‐well plates evenly. Incubate the plate for 15 min under room temperature and perform the measurement in triplicate at the wavelength of 515 nm by using a microplate reader.

#### 2.4.2 Measurement of radical scavenging capabilities by ABTS assay

Add ABTS mother solution and oxidant solution into a 10 ml brown bottle by 1:1. Vortex them evenly and store them under room temperature in the dark for 12 hr before use. The solution was kept stable for 2‐3 days. Use a microplate reader to measure the actual value of A in the equation *A* = *A*_734_ ‐ *A*_0_ and dilute ABTS mother solution with 80% of ethanol until the actual value of A equaling to 0.7 ± 0.05. *A*_0_ is the value of the blank control (absorbance value measured using 80% of ethanol solution without samples).

The standard curve solution was prepared as follows: dilute Trolox standards with 80% of ethanol to 0.15, 0.3, 0.6, 0.9, 1.2, and 1.5 mmol/L.

The measurement was performed in the following procedure: 200 μl ABTS working solution was added into the wells of a 96‐well plates. 10 μl distilled water was added into blank control wells. 10 μl previously prepared Trolox standard solutions at different concentrations were added into standard curve wells. Add 10 μl test samples into sample wells. Slightly stir them to obtain evenly mixed solutions. Incubate the plate under room temperature for 5 min, and then measure the absorbance values at the wavelength of 734 nm using a spectrophotometer. Calculate the total antioxidant capabilities according to the standard curve established based on the absorbance values and concentrations of the standards. If the absorbance of a measured substance falls beyond the standard curve range, dilute or concentrate the sample before measurement. The total antioxidant capability is expressed as the Trolox‐Equivalent Antioxidant Capacity (TEAC).

#### 2.4.3 Measurement of radical scavenging capabilities by FRAP assay

Prepare appropriate amounts of working solutions according to the number of target samples. A volume of 180 μl FRAP working solution is required for each test sample, which comprises 150 μl of TPTZ diluent, 15 μl of TPTZ solution, and 15 μl of buffer. The buffer was added last after the first two solutions were vortexed evenly. The FRAP working solution can be used only after incubation at 37°C, and solution exhaustion within 2 hr is recommended.

The standard curve solution was prepared as follows: Accurately weigh 27.8 mg of FeSO_4_^.^7H_2_O and add water to a fixed volume of 1 ml, which makes 100 mmol/L standard stock solution. Dilute the mother liquor with ethanol to 0.15, 0.3, 0.6, 0.9, 1.2, and 1.5 mmol/L for later use.

Note that the FeSO_4_ solution should be prepared on site because it is unstable and is prone to contact with air to become Fe^3+^ as a result of oxidation. Stop using the solution when it is changed from light green to light yellow.

The measurement was performed as follows: (a) Add 180 μl FRAP working solution to the wells of a 96‐well plates. (b) Add 5 μl distilled water to blank control wells. (c) Add 5 μl prepared FeSO_4_ standard solutions at different concentrations into standard curve wells. Add 5 μl test samples into sample wells. (d) Slightly stir them to obtain evenly mixed solutions. Incubate the plate in a 37°C incubator for 5 min, and then measure the absorbance at 593 nm. (e) Calculate the total antioxidant capabilities according to the standard curve established based on the absorbance values and concentrations of the standards. The total antioxidant capability is represented by the concentration of the FeSO_4_ standard solution.

### 2.5 Statistical analyses

All antioxidant capability tests were performed in triplicate during the entire course, and all statistical results were analyzed by the SAS software.

## 3 RESULTS AND DISCUSSION

### 3.1 Standard curves for different antioxidant assays

**Figure 1:**
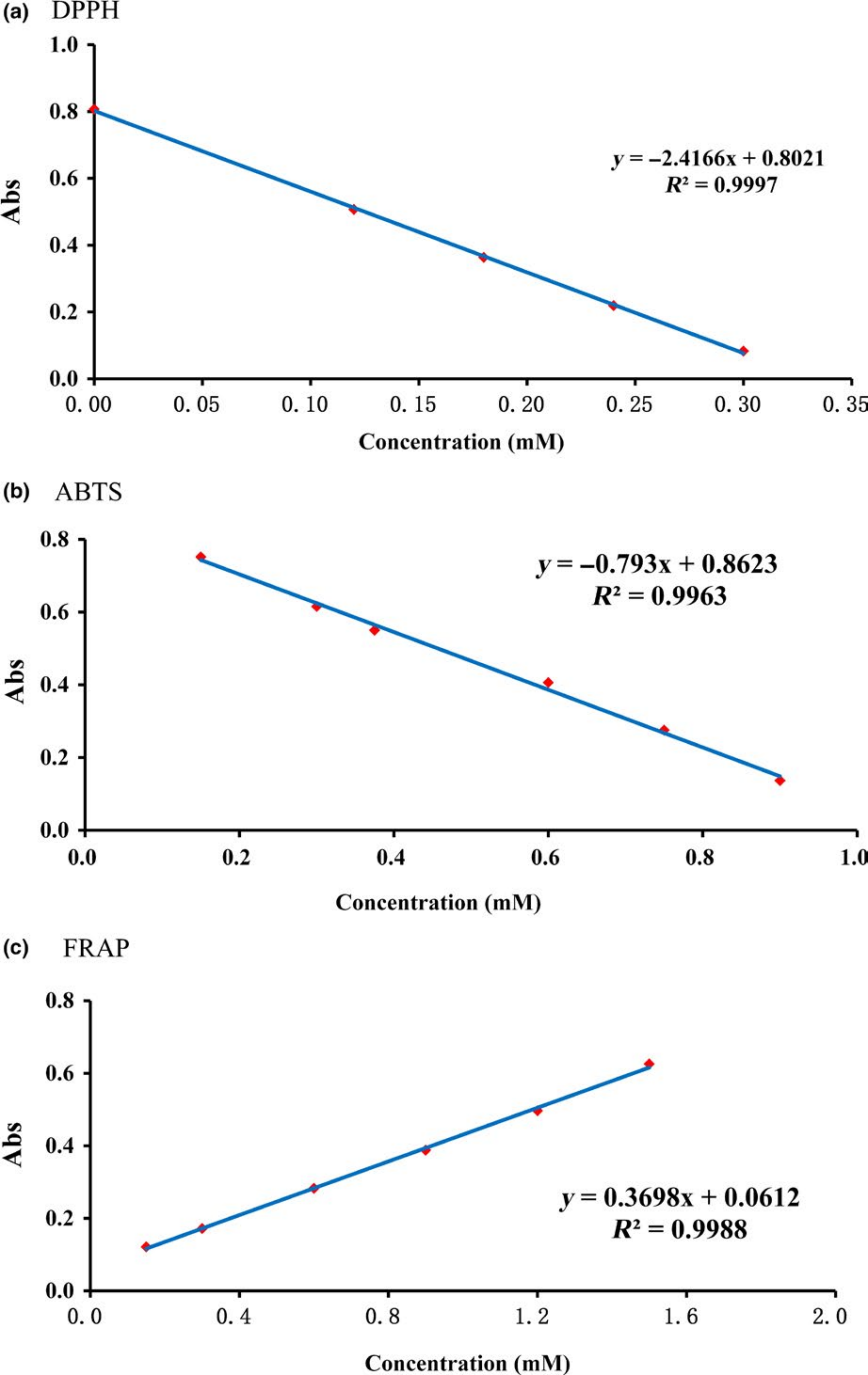
Standard curves of the DPPH, ABTS, and ferric reducing ability of plasma (FRAP) assays (a) DPPH, (b) ABTS, (c) FRAP

The antioxidant capabilities were measured by DPPH, ABTS, and FRAP assays to construct standard curves. The measurements were conducted in triplicate to obtain average values, and the data were sent for processing using Origin 7.5. Then, standard curves were drawn according to the concentrations of standard solutions against the absorbance values within their respective ranges. As shown in Figure 1, excellent linear relationships were expressed by the following equations:

$$\text{DPPH}:y\;=\;-2.416x+0.802,\;R^{2}\;=\;0.999$$

$$\text{ABTS}:y\;=\;-0.793x+0.862,\;R^{2}\;=\;0.996$$

$$\text{FRAP}:y\;=\;0.369x+0.061,\;R^{2}\;=\;0.998$$

### 3.2 Evaluation of the antioxidant capabilities of eleven edible vegetable oils

FR chain reactions involve a series of complicated oxidative reactions such as hydrolysis, oxidation, and polymerization, which eventually lead to rancidity of edible vegetable oils and deteriorated nutritional quality of oils (Johnson & Decker, 2015). In this paper, FR chain reactions based on hydroxyl oxygen radicals, superoxide radicals, and hydrogen peroxide were selected to evaluate the antioxidant functions of vegetable oils. The DPPH, ABTS, and FRAP methods were employed to evaluate the radical scavenging capabilities of the extracts from edible vegetable oils.

**Figure 2:**
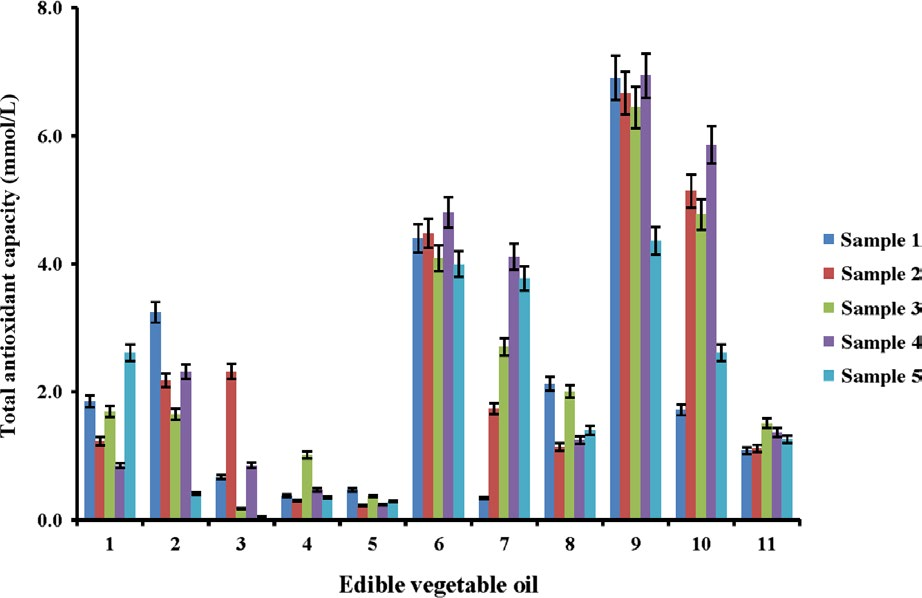
Total antioxidant capacity of different edible oils measured by DPPH assay (*n* = 3)

**Figure 3:**
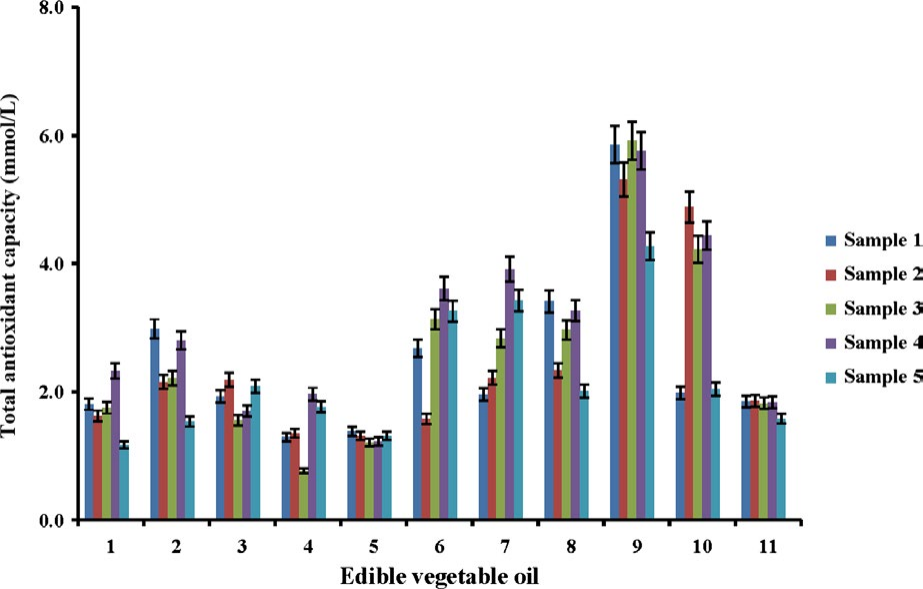
Total antioxidant capacity of different edible oils measured by ABTS assay (*n* = 3)

**Figure 4:**
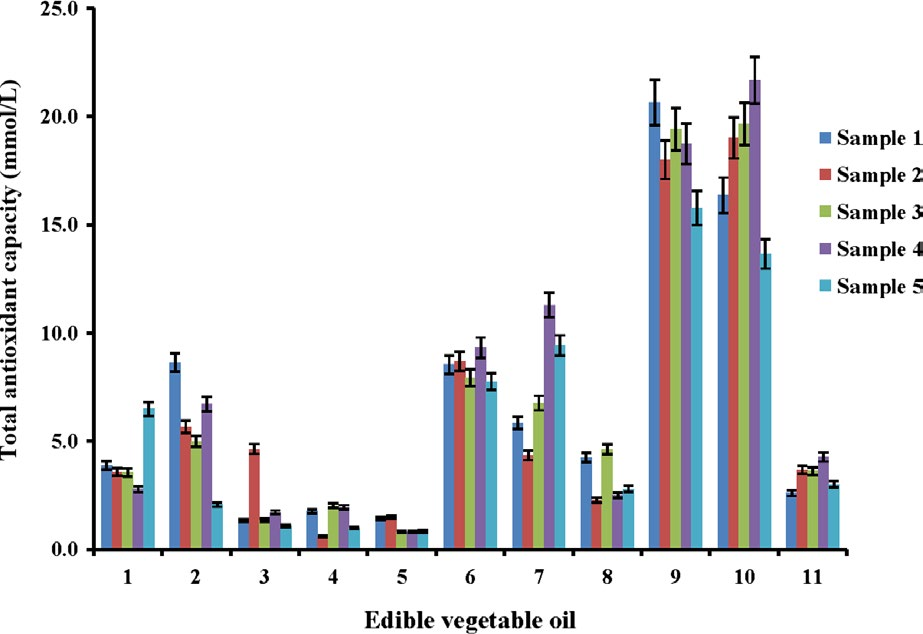
Total antioxidant capacity of different edible oils measured by ferric reducing ability of plasma (FRAP) assay (*n* = 3)

Antioxidant capabilities of different edible vegetable oils including flaxseed oil (1), olive oil (2), grape seed oil (3), corn oil (4), soybean oil (5), sunflower seed oil (6), walnut oil (7), perilla oil (8), rapeseed oil (9), sesame oil (10), and camellia oil (11) were investigated by DPPH, ABTS, and FRAP assays. As shown in Figures 2, 3, 4, their antioxidant capabilities were varied. Despite different antioxidant capabilities measured by these assays, the total antioxidant capabilities of rapeseed oil and sesame oil were the highest, followed by sunflower seed oil, walnut oil, and flaxseed oil. The lowest total antioxidant capabilities were detected in olive oil, grape seed oil, corn oil, camellia oil, and soybean oil. This phenomenon might be attributed to the varied processing techniques and different amounts of antioxidant active compounds in these edible vegetable oils. The edible vegetable oils that had a relatively low antioxidant capability were bought from the market while the oils with superior antioxidant capabilities were all virgin oils prepared in our laboratory. The results indicated that antioxidant components in the oils might be lost during the complex processing procedures. During the bleaching and alkali refining procedures of vegetable oils, a small number of active components may be absorbed by bleaching agents and soap, which decreases their concentrations (Ergönül & Köseoğlu, 2014; Kreps, Vrbiková, & Schmidt, 2015). During the deodorization process, the components such as tocopherols may become decomposed due to high temperature (Rossi, Alamprese, & Ratti, 2007). The test results suggested that suitable refining should minimize the loss of antioxidant components and people should consume virgin oils or edible vegetable oils less processed.

### 3.3 Comparison of radical scavenging capabilities of virgin camellia oils with commercially available refined camellia oils

**Figure 5:**
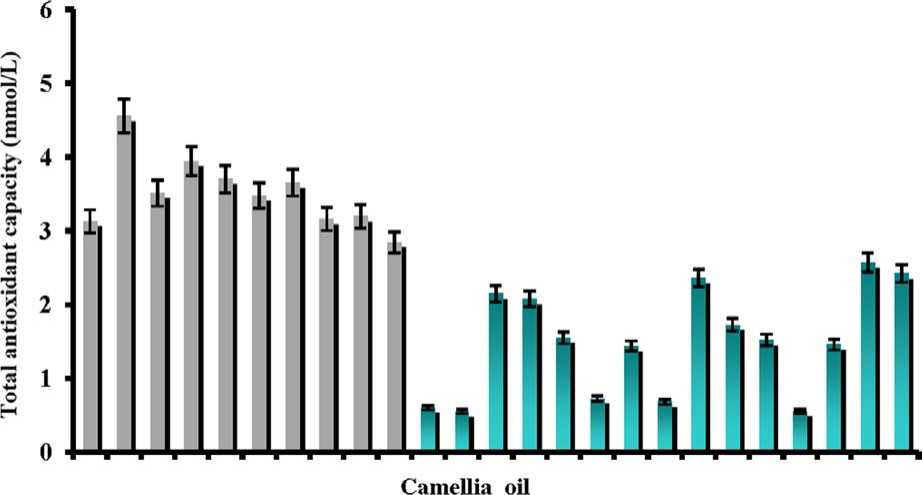
Radical scavenging capabilities of the extracts of the virgin and refined camellia oils (*n* = 3)

Ferric reducing ability of plasma was used to compare the radical scavenging capabilities of the extracts from virgin camellia oils with those of commercially available refined camellia oils. Figure 5 showed the radical scavenging capabilities of the extracts from the virgin camellia oils and commercially available refined camellia oils. As illustrated in this figure, the antioxidant capabilities of the extracts from the two types of camellia oils were greatly different. The refined camellia oils numbered 11, 12, 16, 18, and 22 had excessively low antioxidant capabilities, and the oils numbered 13, 14, 19, 24, and 25 showed similar antioxidant capabilities. This might be caused by their different refining processes and varied amounts of antioxidants added before being sold. Earlier literatures indicated that certain amounts of phytosterols and tocopherols remained in the solid residues after a series of edible vegetable oil processing procedures including pressing, heating, alkali refining, and decoloring (Ferrari, Schulte, Esteves, Brühl, & Mukherjee, 1996; Ghazani & Marangoni, 2013). Some weak acid antioxidant materials such as polyphenols may react with alkali substances during the alkali refining procedure, which decreases the antioxidant material concentrations (Deng, Deng, Hu, Li, & Fan, 2015). Therefore, the antioxidant capabilities of the measured virgin camellia oils were higher than the refined oils due to the oversimplified processing procedures and retention of many antioxidant materials to produce better antioxidant effects for the virgin camellia oils. It indicated that virgin vegetable oils had higher nutritional values than refined vegetable oils.

## 4 CONCLUSIONS

In this study, ABTS, FRAP, and DPPH assays were employed to evaluate the radical scavenging capabilities of unsaponifiable fraction of edible vegetable oil. It was discovered that rapeseed oil, and sesame oil had higher total antioxidant capabilities than other oils. Moreover, the FRAP method was used to compare the radical scavenging capabilities of the extracts from virgin camellia oil with those of commercially available refined camellia oils. The results showed that the antioxidant capabilities of virgin camellia oils were higher than refined camellia oils. Therefore, it is recommended that virgin edible vegetable oils be used rather than refined oils with their radical scavenging capabilities considered.

## CONFLICT OF INTEREST

The authors declare that they do not have any conflict of interest.

## ETHICAL APPROVAL

This study does not involve any human or animal testing.
